# Solvatochromic and Single Crystal Studies of Two Neutral Triarylmethane Dyes with a Quinone Methide Structure

**DOI:** 10.3390/molecules201119724

**Published:** 2015-11-19

**Authors:** Katherine Chulvi, Ana M. Costero, Luis E. Ochando, Salvador Gil, José-Luis Vivancos, Pablo Gaviña

**Affiliations:** Centro de Reconocimiento Molecular y Desarrollo Tecnológico (IDM), Universitat de València-Universidad Politécnica de Valencia, Dr. Moliner, 50, 46100-Burjassot, Valencia, Spain; katherine.chulvi@uv.es (K.C.); luis.e.ochando@uv.es (L.E.O.); salvador.gil@uv.es (S.G.); jvivanco@dpi.upv.es (J.-L.V.)

**Keywords:** triarylmethane dyes, crystal structure, solvatochromic studies

## Abstract

The crystal structure of two neutral triarylmethane dyes with a p-quinone methide core was determined by X-ray diffraction analysis. The spectroscopic characteristics of both compounds in 23 solvents with different polarities or hydrogen-bonding donor (HBD) abilities has been studied as a function of three solvatochromic parameters (*E*_T_(30), π* and α). Both compounds **1** and **2** showed a pronounced bathochromic shift of the main absorption band on increasing solvent polarity and HBD ability. The correlation is better for compound **2** than for compound **1**. The stronger effect and better correlation was observed for compound **2** with the increment of the solvent HBD ability (α parameter).

## 1. Introduction

The development of chromogenic and fluorogenic probes for detecting a large number of target molecules has gained increased interest in recent years [1,2,3,4]. Colorimetric and fluorimetric detection are particularly appealing as they make use of low cost systems capable of performing quantitative analysis with widespread technologies. They also offer the possibility to detect analytes with the “naked-eye”. Triarylmethane dyes are a very important class of commercial dyes. In addition to their traditional applications in industry and in biological sciences [5,6,7], these dyes have recently demonstrated their utility in the design of chemosensors for different species [8,9,10,11] or dye-sensitized solar cells [12]. These dyes exhibit many interesting photochemical and photophysical properties which are directly related to their structures. In addition, their UV-VIS spectra are also dependent on different factors such as changes in pH, concentration, solvent, pressure or temperature [13].

**Chart 1:**
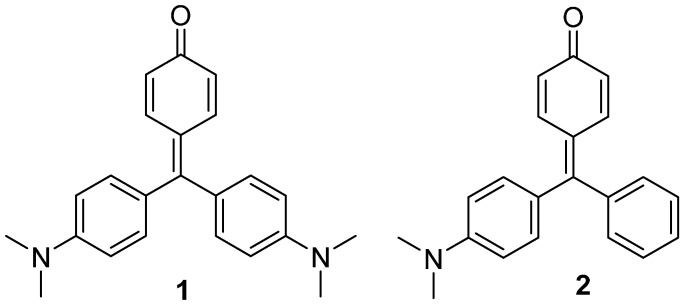
Triaryl methane dyes **1** and **2** with a quinone methide structure.

In our research related to the use of triarylmethane dyes as signaling units in the design of colorimetric probes [8], we have decided to study the changes induced by different solvents in the photophysical properties of compounds **1** and **2** (Chart 1) and to evaluate the influence that the different substitution in the phenyl rings has in these changes. Additionally, the X-ray crystal structures of both compounds have been elucidated.

## 2. Results and Discussion

### 2.1. Synthesis and X-ray Diffraction Studies

**Scheme 1:**
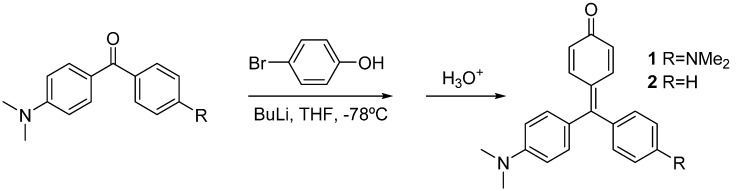
Synthesis of compounds **1** and **2**.

Compounds **1** and **2** were synthesized by lithiation of 4-bromophenol with two equivalents of butyl lithium, and subsequent addition of the Michler’s ketone or 4-dimethylamino benzophenone, respectively. Acidic work-up was used to force dehydration of the carbinols to yield the corresponding dyes [8] (Scheme 1).

**Figure 1:**
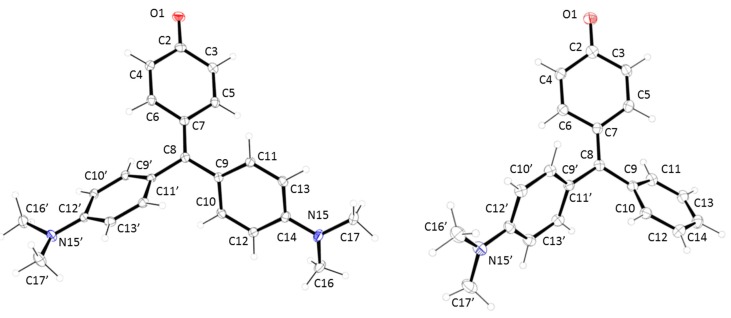
X-ray structures for (**left**) compound **1** and (**right**) compound **2**.

Single crystals of compounds **1** and **2** suitable for X-ray diffraction were obtained by slow evaporation of trichloromethane and ethyl acetate solutions, respectively. There are eight symmetry-independent molecules in the orthorhombic unit cell of compound **1** with Pbca as space group. In the case of compound **2**, the unit cell was monoclicic P2_1_/c with four molecules in the unit cell. Selected geometric characteristics are present in Table 1. Both structures are very similar (see Figure 1), with a clear double bond character in the O(1)-C(2) bond (1.248(3) and 1.249(3) Å for **1** and **2**, respectively). These data indicate that both compounds have a strong *para*-quinone methide structure (Chart 1). This suggestion is corroborated by the short distance of the C(7)-C(8) bond (1.396(3) and 1.381(4) Å for **1** and **2**, respectively). In the same sense, the quinone ring shows the expected values for the simple and double bonds in this type of compounds. The hybridation of C(8) is sp^2^ with bond angles of C(7)-C(8)-C(9), C(7)-C(8)-C(9′) and C(9)-C(8)-C(9′) of 123.25(19)°, 120.0(2)° and 116.71(19)° for compound **1** and 121.4(3)°, 121.7(3)° and 116.8(3)° for compound **2**. On the other hand, a clear contribution of the lone electron pair of the nitrogen atoms in the conjugation system is present, being the ArC-N bond distances similar to these showed by aromatic amines with a planar distribution of the dimethylamine group (see Table 2).

**Table 1:** Selected atomic bond distances and angles for compounds **1** and **2**.

Atomic Bond Distances (Å)	1	2	Angles (°)	1	2
O1-C2	1.248(3)	1.249(3)	C8-C7-C5	123.1(2)	123.4(3)
C2-C4	1.445(3)	1.446(4)	C8-C7-C6	121.4(2)	121.3(3)
C2-C3	1.451(3)	1.451(4)	C7-C8-C9	123.25(19)	121.4(3)
C3-C5	1.384(3)	1.342(4)	C7-C8-C9′	120.0(2)	121.7(3)
C4-C6	1.340(3)	1.346(4)	C9-C8-C9′	116.71(19)	116.8(3)
C5-C7	1.440(3)	1.440(4)			
C6-C7	1.441(3)	1.452(4)			
C7-C8	1.396(3)	1.381(4)			
C8-C9	1.454(3)	1.482(4)			
C8-C9′	1.469(3)	1.466(4)			

**Table 2:** Torsion angles, atomic bond distances and angles for the dimethylamino group in compounds **1** and **2**.

	Angles (°)			Atomic Bond Distances (Å)	Torsion Angles (°)	
	C14′-N15′-C16′	C14′-N15′-C17′	C16′-N15′-C17′	C14′-N15′	C12′-C14′-N15′-C16′	C13′-C14′-N15′-C17′
**1**	118.6(2)	119.8(2)	116.7(2)	1.373(2)	−21.3(3)	5.1(3)
**2**	120.6(3)	121.6(3)	117.7(3)	1.372(4)	−1.5(5)	2.8(5)
	C14-N15-C16	C14-N15-C17	C16-N15-C17	C14-N15	C12-C14-N15-C16	C13-C14-N15-C17
**1**	118.6(2)	121.5(2)	116.1(2)	1.380(3)	13.9(4)	−9.6(4)

In addition, helical rotations are present in the aromatic rings, for both compounds **1** and **2**, which are responsible of the non-planarity in the molecules. Analysing the values of the dihedral angles on the three aromatic rings, it is possible to confirm a propeller like conformation (Table 3) with one ring twisted in the opposite sense to the other two. The torsion angles are lower in the *p*-quinone methide ring (around 20°) than in the phenyl and dimethylaniline rings (around 40°).

**Table 3:** Torsion angles for aromatic rings.

	Torsion Angles (°)		
	C6-C7-C8-C9′	C7-C8-C9-C11	C7-C8-C9′-C11′
**1**	18.1(3)	35.8(3)	−134.6(2)
**2**	−20.4(5)	−40.5(5)	139.2(3)

**Figure 2:**
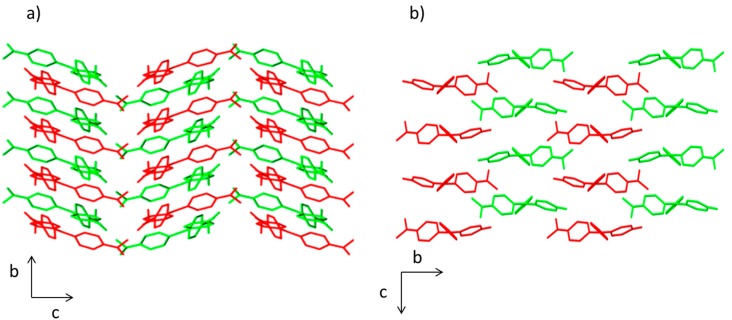
View of the packing diagram along *a* axis for (**a**) compound **1** and (**b**) compound **2**. Hydrogen atoms have been omitted for clarity.

The crystal packing in compounds **1** and **2** is different. In both structures, there is an ABABA three-dimensional disposition. However, a herringbone pattern is present in the compound **1**, whereas brickwork motif is observed in compound **2** (Figure 2). This effect influences the molecular volume being higher for compound **1** (457.63 Å^3^) than for compound **2** (401.23 Å^3^).

### 2.2. Solvatochromic Studies

**Figure 3:**
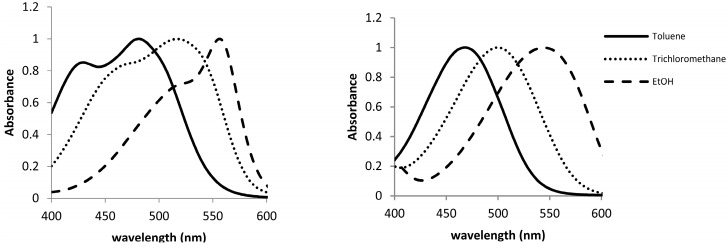
Normalized UV-VIS spectra of 1·10^−5^ mol·L^−1^ solutions of **1** (**left**) and **2** (**right**) in toluene, CHCl_3_, and EtOH.

The UV-VIS spectra of 1·10^−5^ M solutions of compounds **1** and **2** in different solvents were registered at room temperature, in order to study their solvatochromic behavior. As can be seen in Figure 3, the UV-VIS spectra of both compounds show different pattern in toluene. Thus, whereas compound **1** presents a main absorption band at 485 nm with a marked shoulder at 430 nm, compound **2** shows only a band at 470 nm. This behavior is similar to the classical one described in the pioneer publication of Lewis [13] for crystal violet (tris(*p*-dimethylaminophenyl)methylium ion, **CV**) and malachite green (phenyl bis(*p*-dimethylaminophenyl)methylium ion, **MG**) which in some solvents show either two or one absorption bands, respectively.

Since Lewis’s publication suggesting the existence of two isomers in **CV** solutions, a large number of explanations for the shoulder observed in the absorption spectrum of **CV** have been proposed [14], being one of the most accepted that the two bands arise from ground-state absorptions into two neighboring excited states [15]. However, the possibility of an equilibrium between two ground states has also been suggested [16,17,18,19]. On the other hand, it has been also argued that counter anions interacting with the dimethylamino groups of **CV** could be responsible for the changes in the UV-VIS spectra [20]. In our case, as **1** and **2** are neutral compounds no effect of counter anion can be observed and thus the presence of two bands in the UV-visible spectrum of **1** has to be related to the existence of an equilibrium between two ground states or the split of the excited state into two neighboring electronic levels.

When UV-VIS spectra of both compounds (1·10^−5^ mol·L^−1^) in different solvents were registered, the observed tendency for compound **1** was similar to that described for **CV** [21,22] *i.e.*, a bathochromic shift of the overlapped absorption bands on increasing the solvent polarity. Thus, as shown in Figure 3, on changing the solvent from toluene to trichloromethane and ethanol (for the other solvents, see Supplementary Material), a red shift in the wavelengths of maximum absorption bands of compound **1** was observed. This shift was larger for the short-wavelength side of the absorption envelope, diminishing the magnitude of the splitting between the overlapped absorption bands. This fact gives rise to a small resolution in the spectra for the alcoholic solvents and even to a unique band in the case of water. As it was expected, compound **2**, which can be related with **MG**, only shows a band in the UV-spectra, which also experiments with a bathochromic shift on increasing the polarity of the solvent.

Taking into account these results, a more complete study of the solvatochromic properties of compounds **1** and **2** was carried out using 23 solvents with different properties (see Table 4).

The interactions of a solute with solvent molecules can be classified into nonspecific, such as polarity effects, or specific, such as hydrogen bonding. The α and β scales are used to describe the ability of a hydrogen-bond donor (HBD) solvent to donate a proton to the solute or that of a hydrogen-bond acceptor (HBA) solvent to accept a proton from the solute, respectively [23,24]. On the other hand, the solvent polarity scale *E*_T_(30) of Reichardt [25] and the solvent dipolarity/polarizability scale π* of Kamlet *et al.* [23,24] are widely used as nonspecific solvatochromic polarity scales, and also as parameters of linear solvation energy relationships.

**Table 4:** Solvatochromic parameters π*, β and α [23,24] *E*_T_(30) [25] of studied solvents, and λ_max_ (nm) and *E*_T_ (kcal·mol^−1^) of compounds **1** and **2**.

Solvent	Type of Solvent	*E*_T_(30)	π*	β	α	λ_max_(1)	λ_max_(2)	*E*_T_(1)	*E*_T_(2)
Toluene	NHB-weak HBA	33.9	0.54	0.11	0.0	480.5	467	59.50	61.22
Benzene	NHB-weak HBA	34.3	0.59	0.10	0.0	483	469.5	59.20	60.90
Diethylether	HBA	34.5	0.27	0.47	0.0	475	456.5	60.19	62.63
Bromobenzene	NHB-weak HBA	36.6	0.79	0.06	0.0	504	488.5	56.73	58.53
Chlorobenzene	NHB-weak HBA	36.8	0.71	0.07	0.0	503	484.5	56.84	59.01
THF	HBA	37.4	0.58	0.55	0.0	487.5	474.5	58.65	60.26
Ethyl acetate	HBA	38.1	0.55	0.45	0.0	482.5	471	59.26	60.70
Methyl acetate	HBA	38.9	0.60	0.42	0.0	490	474.5	58.35	60.26
Trichloromethane	weak HBD	39.1	0.58	0.0	0.44	515	498.5	55.52	57.35
Pyridine	HBA	40.5	0.87	0.64	0.0	512.5	499	55.79	57.30
Dichloromethane	NHB	40.7	0.82	0.0	0.30	510.5	494	56.01	57.88
2-Butanone	HBA	41.3	0.67	0.48	0.0	503.5	483	56.79	59.20
Acetone	HBA	42.2	0.71	0.48	0.0	501	487	57.07	58.71
*N*,*N*-Dimethylacetamide	HBA	42.9	0.88	0.76	0.0	510	497.5	56.06	57.47
DMF	HBA	43.2	0.88	0.69	0.0	514	500.5	55.62	57.13
2-Methylpropan-2-ol	HBA-D	43.3	0.41	1.01	0.68	541	507.5	52.85	56.34
DMSO	HBA	45.1	1.00	0.76	0.0	525	510.5	54.46	56.06
Acetonitrile	weak HBA	45.6	0.75	0.31	0.19	507.5	491.5	56.34	58.17
1-Butanol	HBA-D	49.7	0.47	0.88	0.79	555.5	537	51.47	53.24
1-Propanol	HBA-D	50.7	0.52	0.78	0.78	555.5	534.5	51.47	53.49
Ethanol	HBA-D	51.9	0.54	0.77	0.83	556	543	51.42	52.65
Methanol	HBA-D	55.4	0.60	0.62	0.93	555	552	51.52	51.80
H_2_O	HBA-D	63.1	1.09	0.18	1.17	572	581	49.98	49.21

HBA = hydrogen bond acceptor; HBD = hydrogen bond donor; HBA-D = hydrogen bond acceptor and donor; NHB = non-hydrogen-bonding solvent.

To study the solvatochromic shifts of main absorption bands of **1** and **2**, as a function of the solute/solvent interactions, plots of the corresponding molar electronic transition energy (*E*_T_(dyes)) values (kcal·mol^−1^) [25] *vs.* the solvatochromic parameters *E*_T_(30), π* and α were obtained.

**Figure 4:**
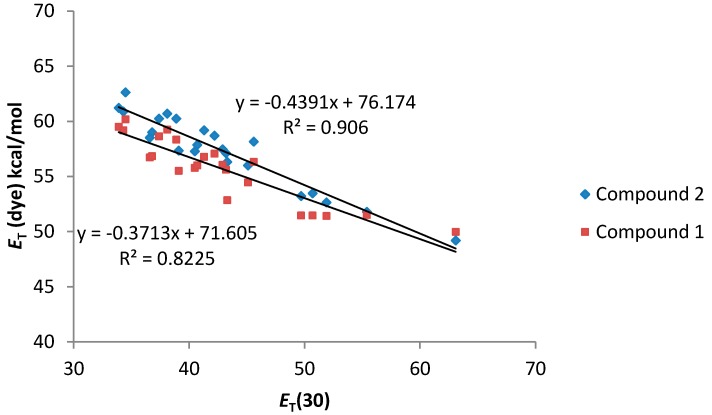
*E*_T_ (dye) values (kcal·mol^−1^) for compounds **1** and **2** *vs.* *E*_T_ (30) solvent polarity parameter.

As can be seen in Figure 4, a good relationship between the *E*_T_ (dye) values and the *E*_T_(30) value of the solvents was observed. In addition, both compounds **1** and **2** present a similar solvatochromic behavior. Even though a gap in the correlations under the transition from the non-polar to alcoholic solutions has been reported for some dyes in the literature [26], compounds **1** and **2** show an almost linear dependence.

**Figure 5:**
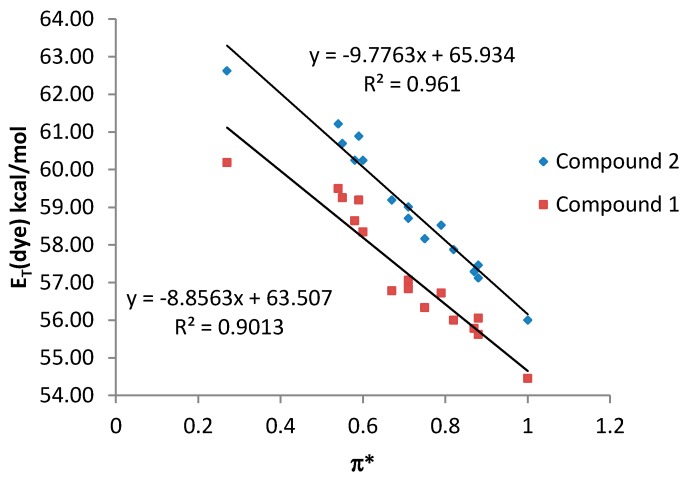
*E*_T_ (dye) values (kcal·mol^−1^) for compounds **1** and **2** *vs.* π* values for NHB and HBA solvents.

By contrast, a poor relationship between the *E*_T_ (dye) values and the π* scale was observed for both compound **1** and **2** when all the studied solvents were considered together (see Supplementary Material). However, a good correlation with the π* parameter was observed when only NHB and HBA solvents are considered, being the results similar for both compounds **1** and **2** (Figure 5).

**Figure 6:**
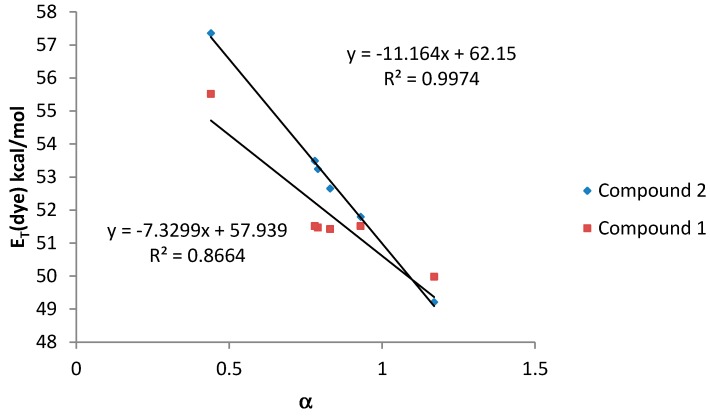
*E*_T_ (dye) values (kcal·mol^−1^) for compounds **1** and **2** *vs.* α values for amphiprotic (HBA-D) solvents.

Finally, when the HBD acidity parameter of the solvent (α scale) was plotted against *E*_T_ (dye) values, a good correlation was also observed for both compounds (Figure 6).

In general, compound **2**, bearing only one dimethylamino group, shows a better correlation with the considered solvatochromic parameters than compound **1**. On the other hand, for compound **2**, the HBD ability of the solvents (parameter α) has a stronger effect on the solvatochromic shift than the nonspecific interactions (*E*_T_(30) and π*). In that sense, the lack of an amino group leads to a much larger solvent effect on the shift in the wavelength of the absorption band probably due to the larger change in the dipolar moment that can be induced in compound **2** after the light absorption. Probably, the presence of two amino groups in compound **1**, lead to a split of the partial dipoles and their vector addition renders a lower dipolar moment. Due to this fact, solvents with high value of α would stabilize more the S_1_ than the S_0_ state and, as a consequence, the energy between both levels would decrease as it was observed.

Finally, a Multiple Linear Regression (MLR) was used in order to find a linear correlation between *E*_T_ (dye) values with the Kamlet-Abboud-Taft solvatochromic parameters (π*, α and β) (Equation (1)). The resulting data matrix contained three variables (*i.e.*, π*, α and β parameters) with 23 rows corresponding to solvents. The adjusted coefficients corresponded to the regression vector obtained (*i.e.*, adjusted coefficients for each compound). Table 5 shows coefficients obtained (C_π*_, C_α_ and C_β_), RMSE (root mean squared error) and correlation coefficient (r) using MLR (see supporting information).

*E*_T_ = *E*_T__(toluene)_ + C_π*_ π* + C_α_ α + C_β_ β (1)

**Table 5:** Adjusted coefficients (C_π*_, C_α_ and C_β_), root mean squared error (RMSE) and correlation coefficients (r) for the Multiple Linear Regression analysis of the absorption λ_max_ of the compounds **1** and **2** with the solvent polarity/polarizability and the acid and base capacity using the Kamlet-Abboud-Taft (π*, α and β) scale.

Compound	C_π*_	C_α_	C_β_	RMSE	r
**1**	−2.177	−6.282	−1.763	0.9344	0.95
**2**	−2.728	−7.192	−0.722	1.1094	0.95

**Figure 7:**
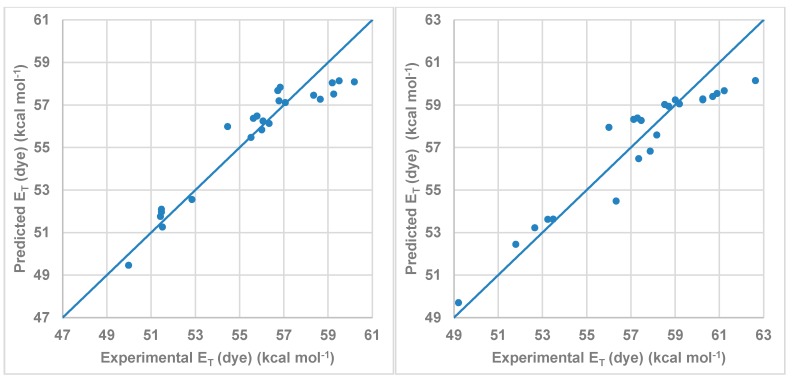
Model obtained using MLR for the Kamlet-Abboud-Taft scale for (**left**) compound **1** and (**right**) for compound **2**.

Figure 7 shows the correlation between the *E*_T_ (dye) values calculated by multi-component linear regression employing the Kamlet-Abboud-Taft-proposed parameters and the experimental values listed in Table 4. As was expected, the dominant coefficient affecting the wavelength shift is that describing the HBD acidity parameter (α) of the solvent followed by the π* parameter. Due to the structure of the studied dye, the influence of the β parameter is small. On the other hand, the C_α_ value was higher for compound **2** than for compound **1** in agreement with the results showed in Figure 6.

## 3. Experimental Section

Suitable crystals for X-ray diffraction studies were grown from trichloromethane (compound **1**) or ethyl acetate (compound **2**) by slow evaporation at 4 °C. The best crystals with approximate dimensions of 0.480 × 0.360 × 0.110 mm^3^ and 0.110 × 0.060 × 0.020 mm^3^ for compounds **1** and **2**, respectively, were selected. Crystals were mounted using nylon CryoLoops of 10 µm diameter (Hampton Research) assisted by Fomblin Y lubricant and supported into the magnetic pin. Crystallographic measurements were made on an Oxford Diffraction Gemini S Ultra equipped with a graphite-monochromated Mo Kα radiation (λ = 0.71073 Å) source and a CCD detector at 120 K. Data were collected to a maximum θ value of 24.996° (99.9% completeness to θ) and 24.999° (99.7% completeness to θ) for **1** and **2**, and then reduced by the Agilent’s CrysAlisPro Software, versions 1.171.34.41 and 1.171.37.35, respectively [27]. The structures were solved and refined using SHELXL-2014 [28], integrated into the WINGX package software [29], by application of direct methods by full matrix least-squares on *F*^2^. The systematic absences clearly pointed to the Pbca space group for compound **1** (orthorhombic, *Z* = 8, *Z*′ = 1) and P2_1_/c for compound **2** (monoclinic, *Z* = 4, *Z*′ = 1). All non–hydrogen atoms were refined anisotropically and the hydrogens attached to N-atoms were located in the difference Fourier map and refined isotropically resulting *R*_int_ = 0.0591 for 3213 data in the compound **1** and *R*_int_ = 0.0616 for 2814 data in the compound **2**. Crystal data are listed in Table 6 and Tables S1–S4. All molecular graphics were created using MERCURY 2.3 [30] and ORTEP-III 1.03 software [29]. Crystallographic data were deposited in Cambridge Crystallographic Data Centre (CCDC 1413997-1413998 contains the supplementary crystallographic data for this paper. These data can be obtained free of charge via http://www.ccdc.cam.ac.uk/conts/retrieving.html (or from the CCDC, 12 Union Road, Cambridge CB2 1EZ, UK; Fax: +44 1223 336033; E-mail: deposit@ccdc.cam.ac.uk).

UV-VIS absorption spectra were performed by using 1 cm path length quartz cuvettes with a Shimadzu UV-2101PC spectrophotometer. The samples were 1 × 10^−5^ M solutions of compounds **1** and **2** in the different studied solvents.

**Table 6:** Crystal data and structure refinement for compounds **1** and **2**.

	Compound 1		Compound 2	
Empirical formula	C_23_H_24_N_2_O		C_21_H_19_NO	
Formula weight	344.44		301.37	
Temperature	120(2) K		120(2) K	
Wavelength	0.71073 Å		0.71073 Å	
Crystal system	Orthorhombic		Monoclinic	
Space group	P b c a		P 2_1_/c	
Unit cell dimensions	a = 17.3098(12) Å	α = 90°	a = 9.8522(8) Å a = 90°	α = 90°
	b = 9.8125(10) Å	β = 90°	b = 17.4626(8) Å	β = 111.916(9)°
	c = 21.5541(19) Å	γ = 90°	c = 10.0550(7) Å	γ = 90°
Volume	3661.0(6) Å^3^		1604.9(2) Å^3^	
Z	8		4	
Density (calculated)	1.250 Mg/m^3^		1.247 Mg/m^3^	
Absorption coefficient	0.077 mm^−1^		0.076 mm^−1^	
F(000)	1472		640	
Crystal size	0.480 × 0.360 × 0.110 mm^3^		0.110 × 0.060 × 0.020 mm^3^	
θ range for data collection	3.019 to 24.996°		3.398 to 24.999°	
Index ranges	−20 ≤ h ≤ 20, −10 ≤ k ≤ 11, −25 ≤ l ≤ 25		−11 ≤ h ≤ 11, −12 ≤ k ≤ 20, −8 ≤ l ≤ 11	
Reflections collected	9574		6095	
Independent reflections	3213 [R(int) = 0.0591]		2814 [R(int) = 0.0616]	
Completeness to θ = 24.996°	99.9%		99.7%	
Refinement method	Full-matrix least-squares on F^2^		Full-matrix least-squares on *F*^2^	
Data/restraints/parameters	3213/0/332		2814/0/285	
Goodness-of-fit on *F*^2^	1.026		0.999	
Final R indices [I > 2sigma(I)]	R1 = 0.0529, wR2 = 0.0843		R1 = 0.0615, wR2 = 0.0945	
R indices (all data)	R1 = 0.0901, wR2 = 0.1027		R1 = 0.1312, wR2 = 0.1287	
Extinction coefficient	0.0020(2)		0.0040(7)	
Largest diff. peak and hole	0.194 and −0.237 e·Å^−3^		0.228 and −0.207 e·Å^−3^	

## 4. Conclusions

The crystal structures of two new triarylmethane derivatives have been elucidated. The solvatochromic properties of both compounds have been studied in front of 23 different solvents. The shift of the main absorption band as a function of different parameters (*E*_T_(30), π*, α) has been considered. Both compounds **1** and **2** showed a pronounced bathochromic shift of the main absorption band on increasing solvent polarity and HBD ability. The correlation is better for compound **2** than for compound **1**. The stronger effect and the better correlation was observed for compound **2** with the increment of the solvent HBD ability (α parameter) in amphiprotic solvents. Multiparameter approach applied to the experimental using the Kamlet-Abboud-Taft solvatochromic parameters shows good correlations between the theoretical and the experimental data. These results suggest that compounds **1** and **2** could be used as solvatochromic dyes for new solvent polarity scale.

## Supplementary Materials

Supplementary materials can be accessed at: http://www.mdpi.com/1420-3049/20/11/19724/s1.
