# Supplementary material for: Solvatochromic and Single Crystal Studies of Two Neutral Triarylmethane Dyes with a Quinone Methide Structure
Source: Molecules. 2015 Nov 19;20(11):20688–98. doi: 10.3390/molecules201119724 (PMC6332080; doi:10.3390/molecules201119724)
Supplement: Supplementary file 1 [file molecules-20-19724-s001.pdf]

Supplementary Material: Solvatochromic and Single Crystal Studies of Two Neutral Triarylmethane Dyes with a Quinone Methide Structure

Katherine Chulvi, Ana M. Costero, Luis E. Ochando, Salvador Gil, José-Luis Vivancos and Pablo Gaviña

General Procedures

All reagents were commercially available, and were used without purification. Silica gel 60 F254 (Merck) plates were used for TLC. Tetrahydrofurane was distilled over Na prior to use. The ^{1}H- and ^{13}C-NMR spectra were recorded using a Bruker DRX-500 spectrometer (500 MHz for ^{1}H and 126 MHz for ^{13}C) and Bruker Avance 400 MHz (400 MHz for ^{1}H and 100 MHz for ^{13}C). UV-Vis measurements were performed using 1 cm path length quartz cuvettes and in a Shimadzu UV-2101PC spectrophotometer.

Compound 1

Compound **1** was synthesized from *p*-bromophenol, 4,4′-bis(dimethylamino)benzophenone and butyllithium in dry THF as previously reported [1].

^{1}H-NMR (400 MHz, $CDCl_3$) δ 7.33 (d, *J* = 9.0 Hz, 4H), 7.20 (d, *J* = 8.9 Hz, 2H), 7.14 (d, *J* = 8.5 Hz, 2H), 6.82 (d, *J* = 9.3 Hz, 4H), 3.26 (s, 12H). ^{13}C NMR (100 MHz, $CDCl_3$) δ 180.29, 156.27, 140.61, 138.97, 126.85, 117.73, 117.12, 114.84, 112.62, 40.47.

Compound 2

Compound **2** was synthesized from *p*-bromophenol, BuLi and 4-dimethylaminobenzophenone in dry THF under argon atmosphere as previously reported [1].

^{1}H-NMR (500 MHz, CD_2Cl_2) δ 7.58–7.53 (m, 2H), 7.51–7.46 (m, 3H), 7.31 (d, *J* = 9.8 Hz, 2H), 7.18 (d, *J* = 8.9 Hz, 2H), 6.75 (d, *J* = 8.9 Hz, 2H), 6.41 (d, *J* = 9.8 Hz, 2H), 3.11 (s, 6H). ^{13}C-NMR (126 MHz, CD_2Cl_2) δ 186.32, 163.08, 152.21, 140.80, 140.03, 135.13, 132.66, 130.03, 127.97, 127.47, 127.04, 127.03, 111.03, 39.88.

Crystallographic Data for Compounds 1 and 2

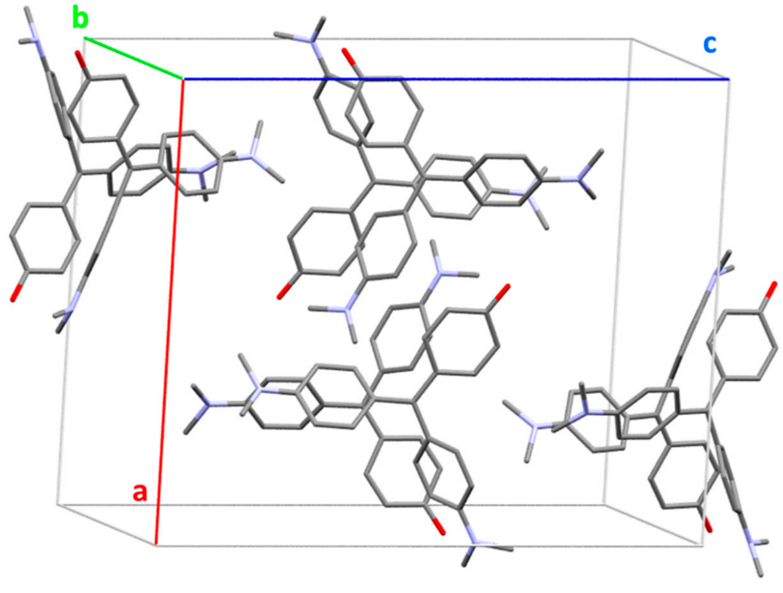

Figure S1. Unit cell of compound **1**.

Table S1. Atomic coordinates (×10[4]) and equivalent isotropic displacement parameters (Å^2 × 10^3) for compound **1**. U(eq) is defined as one third of the trace of the orthogonalized U^{ij} tensor.

	**x**	**y**	**z**	**U(eq)**
C(2)	−215(1)	7482(2)	1258(1)	25(1)
C(3)	−190(1)	7274(2)	592(1)	26(1)
C(4)	−960(1)	7262(3)	1544(1)	26(1)
C(5)	−822(1)	6938(2)	259(1)	24(1)
C(6)	−1588(1)	6955(2)	1208(1)	24(1)
C(7)	−1567(1)	6766(2)	545(1)	21(1)
C(8)	−2234(1)	6451(2)	211(1)	20(1)
C(9)	−2220(1)	5919(2)	−419(1)	20(1)
C(9′)	−2998(1)	6697(2)	485(1)	19(1)
C(10′)	−3176(1)	7917(3)	787(1)	22(1)
C(10)	−2784(1)	6322(2)	−848(1)	23(1)
C(11′)	−3585(1)	5721(3)	452(1)	23(1)
C(11)	−1655(1)	5013(2)	−636(1)	22(1)
C(12′)	−3885(1)	8152(3)	1052(1)	22(1)
C(12)	−2756(1)	5952(3)	−1463(1)	24(1)
C(13)	−1631(1)	4595(2)	−1246(1)	23(1)
C(13′)	−4294(1)	5933(3)	728(1)	24(1)
C(14′)	−4464(1)	7154(2)	1041(1)	22(1)
C(14)	−2165(1)	5099(2)	−1682(1)	24(1)
C(17′)	−5738(2)	6296(3)	1339(2)	42(1)
C(16)	−2578(2)	5551(3)	−2741(1)	35(1)
C(17)	−1459(2)	4058(3)	−2558(1)	33(1)
C(16′)	−5218(2)	8417(3)	1796(1)	36(1)
N(15)	−2122(1)	4770(2)	−2304(1)	31(1)
N(15′)	−5166(1)	7381(2)	1319(1)	28(1)
O(1)	366(1)	7827(2)	1563(1)	34(1)

Table S2. Bond lengths [Å] and angles [°] for compound **1**.

C(2)-O(1)	1.248(3)
C(2)-C(4)	1.445(3)
C(2)-C(3)	1.451(3)
C(3)-C(5)	1.348(3)
C(4)-C(6)	1.340(3)
C(5)-C(7)	1.440(3)
C(6)-C(7)	1.441(3)
C(7)-C(8)	1.396(3)
C(8)-C(9)	1.454(3)
C(8)-C(9′)	1.469(3)
C(9)-C(10)	1.401(3)
C(9)-C(11)	1.403(3)
C(9′)-C(10′)	1.396(3)
C(9′)-C(11′)	1.398(3)
C(10′)-C(12′)	1.374(3)
C(10)-C(12)	1.374(3)
C(11′)-C(13′)	1.380(3)
C(11)-C(13)	1.378(3)
C(12′)-C(14′)	1.402(3)
C(12)-C(14)	1.404(3)

C(13)-C(14)	1.408(3)
C(13′)-C(14′)	1.406(3)
C(14′)-N(15′)	1.373(3)
C(14)-N(15)	1.380(3)
C(17′)-N(15′)	1.455(3)
C(16)-N(15)	1.449(3)
C(17)-N(15)	1.450(3)
C(16′)-N(15′)	1.449(3)
O(1)-C(2)-C(4)	122.3(2)
O(1)-C(2)-C(3)	122.5(2)
C(4)-C(2)-C(3)	115.3(2)
C(5)-C(3)-C(2)	122.5(2)
C(6)-C(4)-C(2)	121.8(2)
C(3)-C(5)-C(7)	121.9(2)
C(4)-C(6)-C(7)	123.0(2)
C(8)-C(7)-C(5)	123.1(2)
C(8)-C(7)-C(6)	121.4(2)
C(5)-C(7)-C(6)	115.5(2)
C(7)-C(8)-C(9)	123.25(19)
C(7)-C(8)-C(9′)	120.0(2)
C(9)-C(8)-C(9′)	116.71(19)
C(10)-C(9)-C(11)	116.4(2)
C(10)-C(9)-C(8)	120.2(2)
C(11)-C(9)-C(8)	123.4(2)
C(10′)-C(9′)-C(11′)	116.8(2)
C(10′)-C(9′)-C(8)	121.8(2)
C(11′)-C(9′)-C(8)	121.4(2)
C(12′)-C(10′)-C(9′)	122.3(2)
C(12)-C(10)-C(9)	122.5(2)
C(13′)-C(11′)-C(9′)	121.4(2)
C(13)-C(11)-C(9)	121.9(2)
C(10′)-C(12′)-C(14′)	121.0(2)
C(10)-C(12)-C(14)	120.5(2)
C(11)-C(13)-C(14)	120.8(2)
C(11′)-C(13′)-C(14′)	121.4(2)
N(15′)-C(14′)-C(12′)	120.8(2)
N(15′)-C(14′)-C(13′)	122.2(2)
C(12′)-C(14′)-C(13′)	117.0(2)
N(15)-C(14)-C(12)	120.4(2)
N(15)-C(14)-C(13)	122.1(2)
C(12)-C(14)-C(13)	117.6(2)
C(14)-N(15)-C(16)	118.6(2)
C(14)-N(15)-C(17)	121.5(2)
C(16)-N(15)-C(17)	116.1(2)
C(14′)-N(15′)-C(16′)	118.6(2)
C(14′)-N(15′)-C(17′)	119.8(2)
C(16′)-N(15′)-C(17′)	116.7(2)

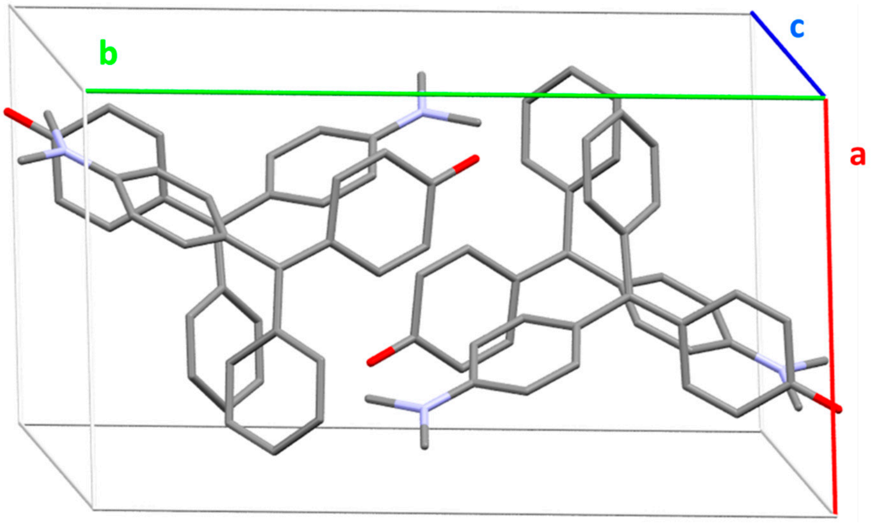

Figure S2. Unit cell of compound **2**.

Table S3. Atomic coordinates (×10⁴) and equivalent isotropic displacement parameters (Å² × 10³) for compound **2**. U(eq) is defined as one third of the trace of the orthogonalized U^{ij} tensor.

	x	y	z	U(eq)
C(2)	2423(4)	−207(2)	6382(4)	34(1)
C(3)	3985(4)	−271(2)	6716(4)	34(1)
C(4)	1573(4)	−890(2)	5824(4)	33(1)
C(5)	4600(4)	−934(2)	6564(4)	33(1)
C(6)	2206(4)	−1556(2)	5707(4)	31(1)
C(7)	3774(3)	−1629(2)	6088(3)	27(1)
C(8)	4407(3)	−2328(2)	6026(3)	25(1)
C(9)	5919(3)	−2380(2)	6056(3)	27(1)
C(9′)	3663(3)	−3052(2)	6044(3)	27(1)
C(10)	6865(4)	−2940(2)	6886(4)	31(1)
C(10′)	2955(3)	−3165(2)	7006(4)	29(1)
C(11)	6460(4)	−1864(2)	5305(4)	32(1)
C(11′)	3687(4)	−3679(2)	5167(4)	32(1)
C(12′)	2324(4)	−3854(2)	7120(4)	30(1)
C(12)	8326(4)	−2957(2)	7023(4)	36(1)
C(13′)	3020(4)	−4365(2)	5233(4)	33(1)
C(13)	7905(4)	−1885(2)	5434(4)	36(1)
C(14)	8854(4)	−2422(2)	6316(4)	36(1)
C(14′)	2338(3)	−4477(2)	6219(4)	31(1)
C(16′)	1057(5)	−5270(2)	7401(5)	46(1)
C(17′)	1781(5)	−5821(2)	5484(5)	47(1)
N(15′)	1713(3)	−5163(1)	6333(3)	40(1)
O(1)	1852(2)	404(1)	6561(3)	43(1)

Table S4. Bond lengths [Å] and angles [°] for compound **2**.

C(2)-O(1)	1.249(3)
C(2)-C(4)	1.446(4)
C(2)-C(3)	1.451(4)
C(3)-C(5)	1.342(4)
C(4)-C(6)	1.346(4)
C(5)-C(7)	1.440(4)
C(6)-C(7)	1.452(4)

C(7)-C(8)	1.381(4)
C(8)-C(9′)	1.466(4)
C(8)-C(9)	1.482(4)
C(9)-C(10)	1.393(4)
C(9)-C(11)	1.403(4)
C(9′)-C(10′)	1.401(4)
C(9′)-C(11′)	1.411(4)
C(10)-C(12)	1.393(4)
C(10′)-C(12′)	1.380(4)
C(11)-C(13)	1.381(4)
C(11′)-C(13′)	1.380(4)
C(12′)-C(14′)	1.418(4)
C(12)-C(14)	1.387(5)
C(13′)-C(14′)	1.404(5)
C(13)-C(14)	1.386(5)
C(14′)-N(15′)	1.372(4)
C(16′)-N(15′)	1.457(5)
C(17′)-N(15′)	1.447(5)
O(1)-C(2)-C(4)	122.2(3)
O(1)-C(2)-C(3)	121.9(3)
C(4)-C(2)-C(3)	115.9(3)
C(5)-C(3)-C(2)	121.7(3)
C(6)-C(4)-C(2)	121.8(3)
C(3)-C(5)-C(7)	122.8(3)
C(4)-C(6)-C(7)	122.4(3)
C(8)-C(7)-C(5)	123.4(3)
C(8)-C(7)-C(6)	121.3(3)
C(5)-C(7)-C(6)	115.3(3)
C(7)-C(8)-C(9′)	121.7(3)
C(7)-C(8)-C(9)	121.4(3)
C(9′)-C(8)-C(9)	116.8(3)
C(10)-C(9)-C(11)	118.0(3)
C(10)-C(9)-C(8)	119.9(3)
C(11)-C(9)-C(8)	122.1(3)
C(10′)-C(9′)-C(11′)	116.9(3)
C(10′)-C(9′)-C(8)	120.6(3)
C(11′)-C(9′)-C(8)	122.4(3)
C(9)-C(10)-C(12)	120.7(3)
C(12′)-C(10′)-C(9′)	122.4(3)
C(13)-C(11)-C(9)	121.2(3)
C(13′)-C(11′)-C(9′)	121.6(3)
C(10′)-C(12′)-C(14′)	120.2(3)
C(14)-C(12)-C(10)	120.4(3)
C(11′)-C(13′)-C(14′)	121.1(3)
C(11)-C(13)-C(14)	120.2(3)
C(13)-C(14)-C(12)	119.4(4)
N(15′)-C(14′)-C(13′)	121.9(3)
N(15′)-C(14′)-C(12′)	120.3(3)
C(13′)-C(14′)-C(12′)	117.8(3)
C(14′)-N(15′)-C(17′)	121.6(3)
C(14′)-N(15′)-C(16′)	120.6(3)
C(17′)-N(15′)-C(16′)	117.7(3)

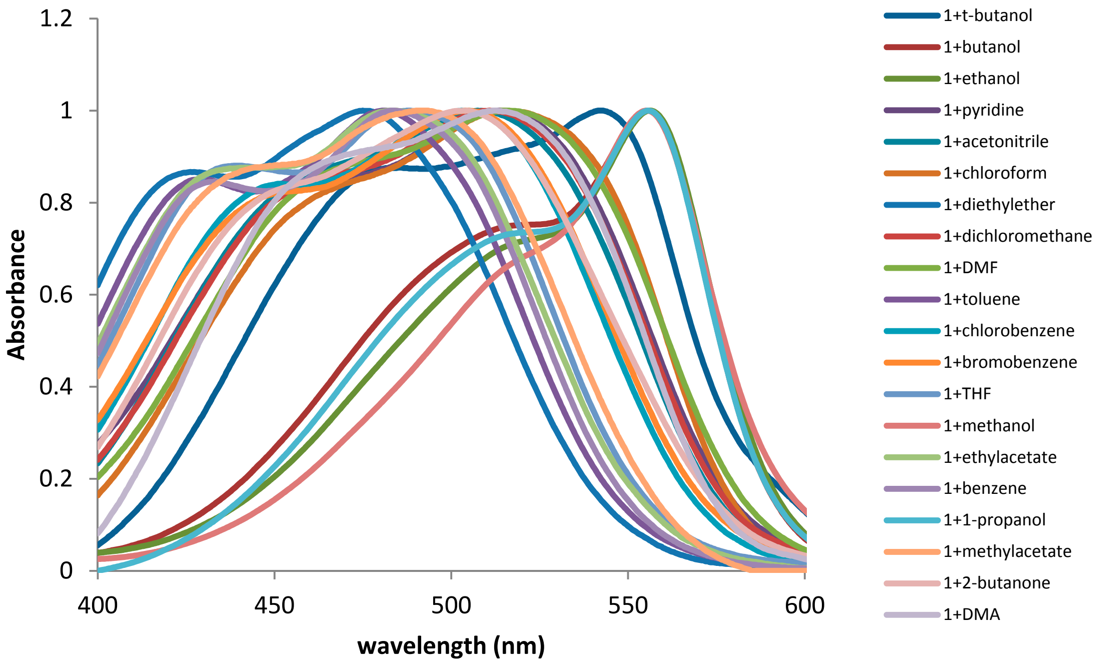

Figure S3. Normalized UV-Vis spectra of compound **1** in the different studied solvents.

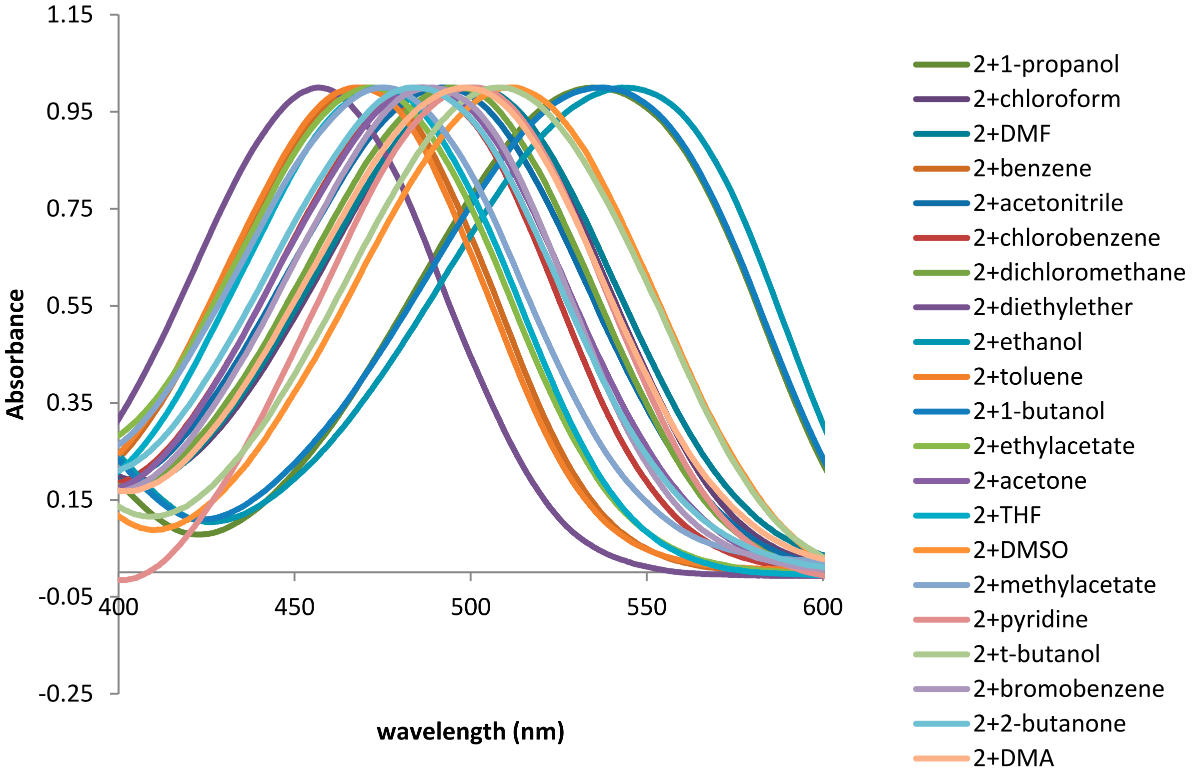

Figure S4. Normalized UV-Vis spectra of compound **2** in the different studied solvents.

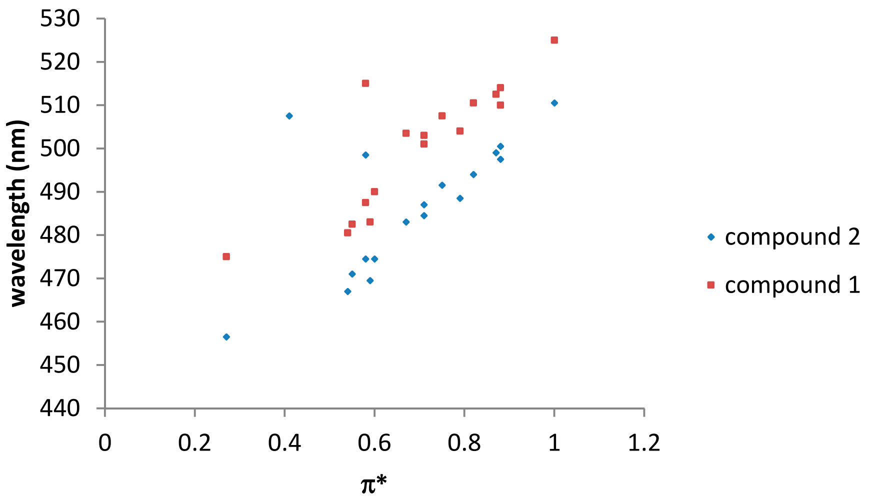

Figure S5. λ_{max} values for compounds **1** and **2** *vs.* π^* values for all the studied solvents.

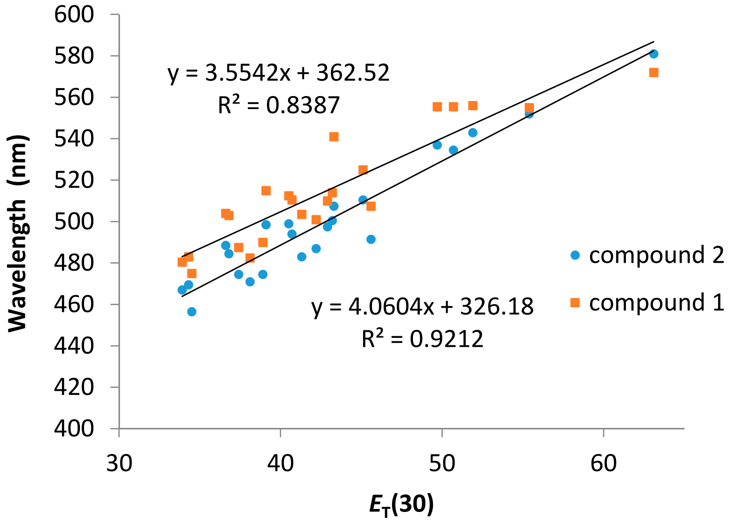

Figure S6. λ_{max} values for compounds **1** and **2** *vs.* solvent polarity.

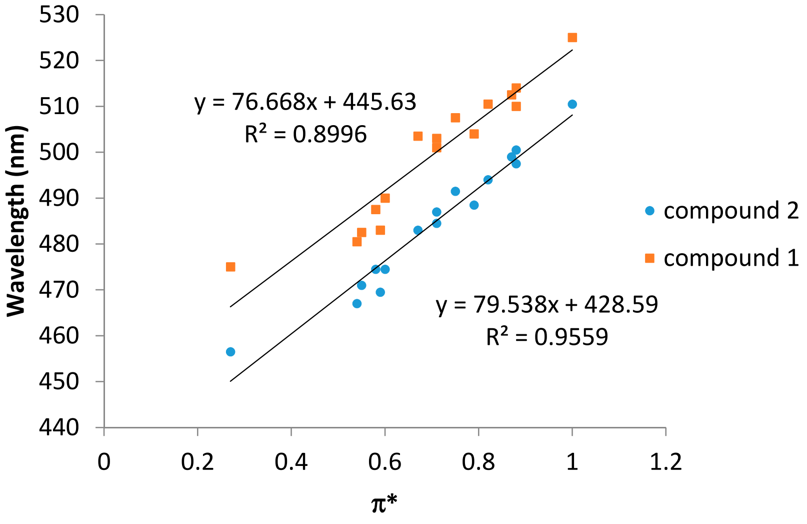

Figure S7. λ_{max} values (Kcal/mol) for compounds **1** and **2** *vs.* π^*.

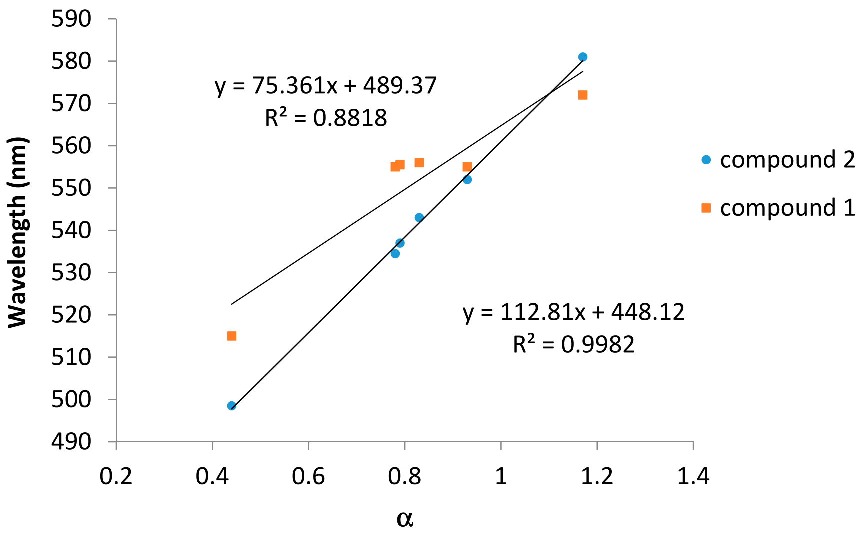

Figure S8. λ_{max} values (Kcal/mol) for compounds **1** and **2** *vs.* α.

Statistical Analysis

Most of the regression models assumes that a regression vector **b** can be used to determine a property of the system **y** from the measured variables **x** (a row vector, such as a spectrum). In this case we can write the model as:

$$\mathbf{x}\,\mathbf{b} = \mathbf{y} \qquad \text{(S1)}$$

The regression vector **b** is determined using a collection of measurements **X** and the known values of the property of interest, **y**. Thus, **b** is estimated from

$$\mathbf{b} = \mathbf{X}^{+}\,\mathbf{y} \qquad \text{(S2)}$$

To determine the pseudoinverse, Multiple Linear Regression (MLR) was used. In this case, X^{+} is defined by

$$\mathbf{X}^{+} = (\mathbf{X}^{T}\mathbf{X})^{-1}\mathbf{X}^{T} \qquad \text{(S3)}$$

All the analyses were performed using the MATLAB® PLS Tool-box (Eigenvector Research, Inc.). MLR was carried out without preprocessing. As a measurement of the fitting of the model the root mean squared error (RMSE) and the correlation (r) were obtained.

Taft Models

The resulting data matrix contained 3 variables (*i.e.*, π^*, α and β parameters) which 23 rows corresponding to solvents. The adjusted coefficients corresponded to the regression vector obtained (*i.e.*, adjusted coefficients for each compound).

The sum of normalized squared scores, known as Hotelling's T^2 statistic, is a measure of the variation in each sample within the model. In Figure S9 is shown the Hotelling's T^2 statistic for Taft Model for the compound **1** for each solvent. Water shows a high value that is a candidate to be an outlier for the model. Figure S10 shows the prediction for the ET parameter for Taft Model for the compound **1**. It can be seen a good correlation which shows a good fitting for the model.

Selectivity Ratio for the variables using Taft Model for the compound **1** were obtained (Figure S11). In Figure S11 a high selectivity for SA parameter is shown.

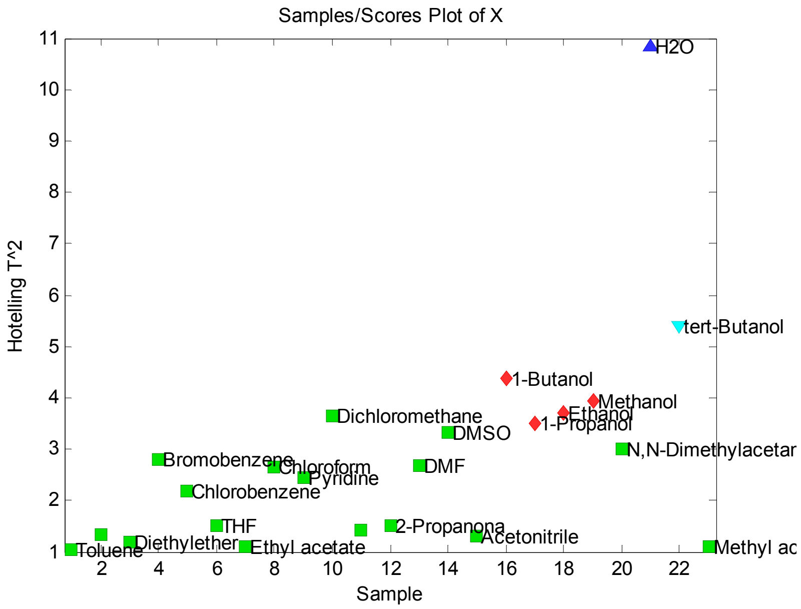

Figure S9. Hotelling (T^2) from samples for MLR using Taft Model for the compound **1**.

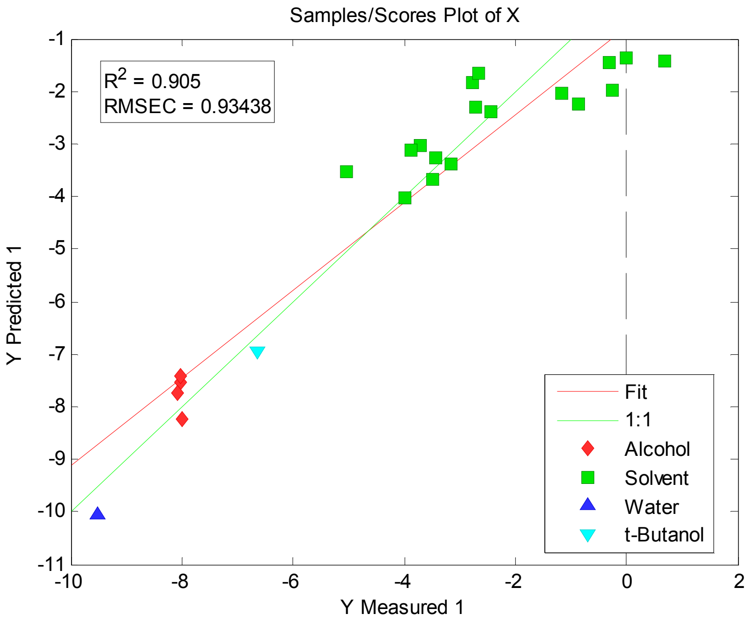

Figure S10. ET Predicted *vs.* ET obtained experimental using Taft Model for the compound **1**.

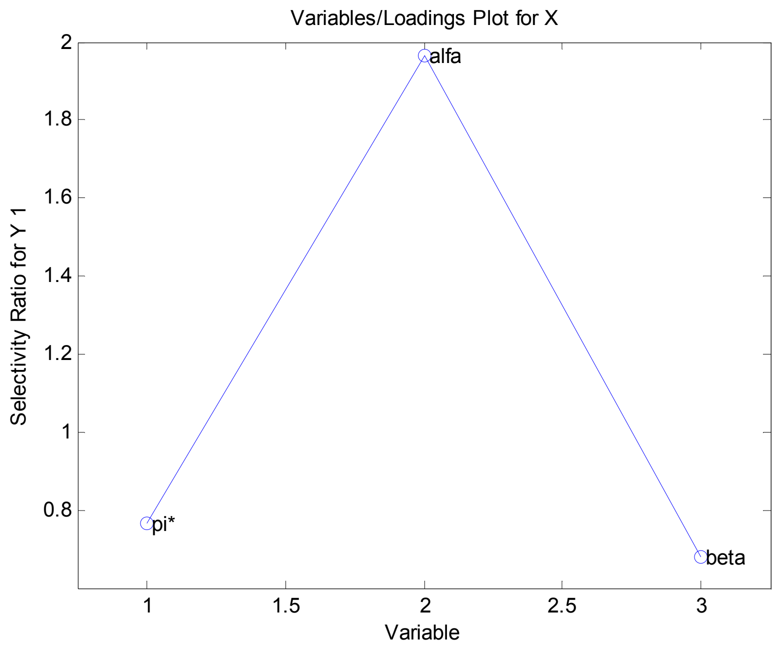

Figure S11. Selectivity Ratio for the variables using Taft Model for the compound **1**.

In Figure S12 is shown the Hotelling's T^2 statistic for Taft Model for the compound **2** for each solvent. Water shows a high value that is a candidate to be an outlier for the model. Figure S13 shows the prediction for the ET parameter for Taft Model for the compound **1**. It can be seen a good correlation which shows a good fitting for the model.

Selectivity Ratio for the variables using Taft Model for the compound **2** was obtained (Figure S14). Figure S14 shows for SA parameter higher selectivity than others parameters.

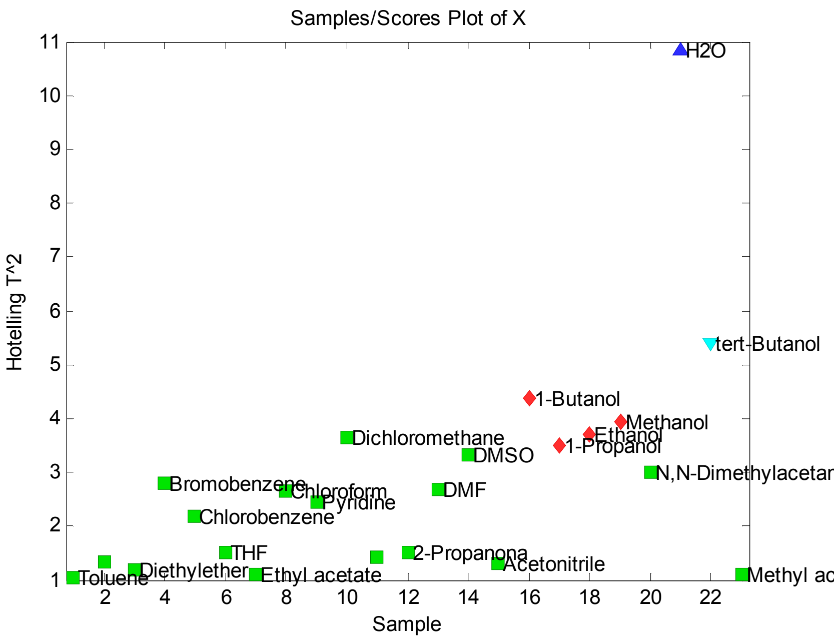

Figure S12. Hotelling (T^2) from samples for MLR using Taft Model for the compound **2**.

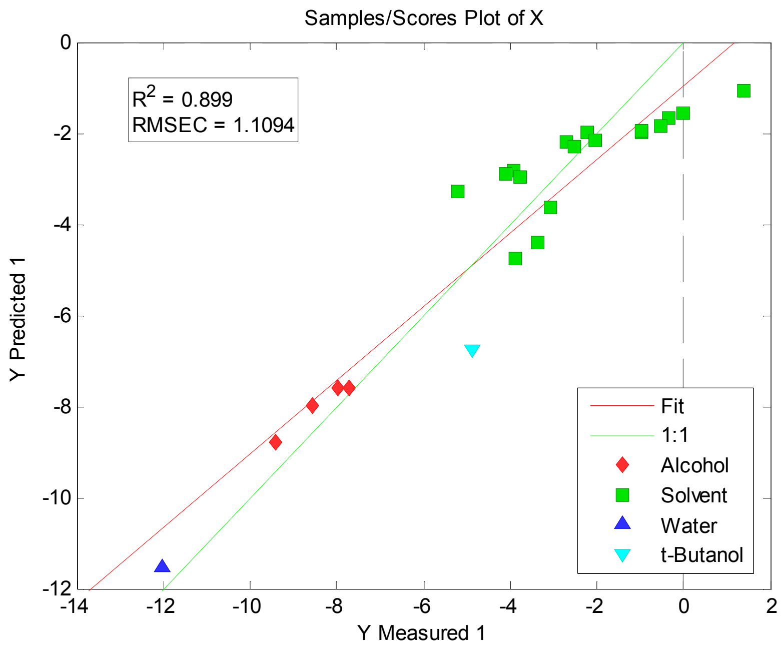

Figure S13. ET Predicted *vs.* ET obtained experimental using Taft Model for the compound **2**.

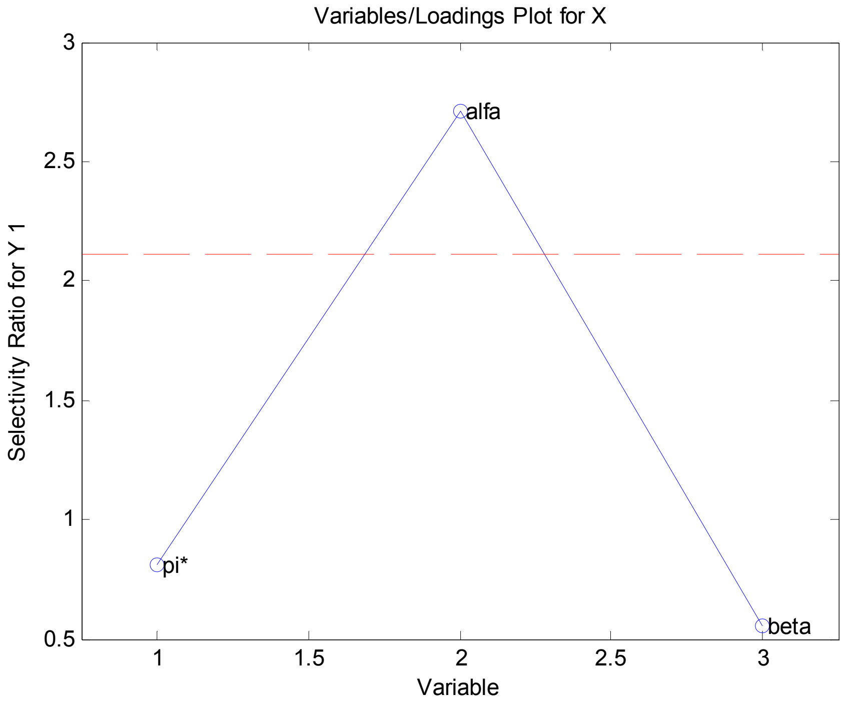

Figure S14. Selectivity Ratio for the variables using Taft Model for the compound **2**.

Reference

1. Gotor, R.; Costero, A.M.; Gil, S.; Parra, M.; Martínez-Máñez, R.; Sancenón, F.; Gaviña, P. Selective and sensitive chromogenic detection of cyanide and HCN in solution and in gas phase. *Chem. Commun.* **2013**, 5669–5671.
